# Immigrants from conflict-zone countries: an observational comparison study of obstetric outcomes in a low-risk maternity ward in Norway

**DOI:** 10.1186/s12884-015-0603-3

**Published:** 2015-08-05

**Authors:** Kjersti S. Bakken, Ola H. Skjeldal, Babill Stray-Pedersen

**Affiliations:** Department of Gynecology and Obstetrics, Baerum Hospital, Vestre Viken Hospital Trust, P.O. Box 800, 3004 Drammen, Norway; Gillberg Neuropsychiatry Centre, Sahlgrenska Academy, University of Gothenburg, Kungsgatan 12, 411 19 Göteborg, Sweden; University of Oslo, Faculty of Medicine, Institution of Clinical Medicine, P.O. Box 1171, Blindern, 0318 Oslo Norway; Women and Children’s Division and Norwegian Resource Centre for Women’s Health, Rikshospitalet, Oslo University Hospital, Sognsvannsveien 20, 0027 Oslo, Norway

## Abstract

**Background:**

Immigrants have higher risks for some adverse obstetric outcomes. Furthermore, refugees are reported to be the most vulnerable group. This study compared obstetric outcomes between immigrant women originating from conflict-zone countries and ethnic Norwegians who gave birth in a low-risk setting.

**Methods:**

This was a population-based study linking the Medical Birth Registry of Norway to Statistics Norway. The study included the first registered birth during the study period of women from Somalia (*n* = 278), Iraq (*n* = 166), Afghanistan (*n* = 71), and Kosovo (*n* = 67) and ethnic Norwegians (*n* = 6826) at Baerum Hospital from 2006–2010. Background characteristics and obstetric outcomes of each immigrant group were compared with ethnic Norwegians with respect to proportions and risks calculated by logistic regression models.

**Results:**

In total, 7408 women and their births were analyzed. Women from Somalia were most at risk for adverse obstetric outcomes. Compared with ethnic Norwegians, they had increased odds ratios (OR) for emergency cesarean section (OR 1.81, CI 1.17–2.80), postterm birth (OR 1.93, CI 1.29–2.90), meconium-stained liquor (OR 2.39, CI 1.76–3.25), and having a small-for-gestational-age infant (OR 3.97, CI 2.73–5.77). They had a reduced OR for having epidural analgesia (OR 0.40, CI 0.28–0.56) and a large-for-gestational-age infant (OR 0.32, CI 0.16–0.64). Women from Iraq and Afghanistan had increased risk of having a small-for-gestational-age infant with OR of 2.21 (CI 1.36–3.60) and 2.77 (CI 1.42–5.39), respectively. Iraqi women also had reduced odds ratio of having a large-for-gestational-age infant (OR 0.35, CI 0.15–0.83). Women from Kosovo did not differ from ethnic Norwegians in any of the outcomes we tested.

**Conclusions:**

Even in our low-risk maternity ward, women originating from Somalia were at the greatest risk for adverse obstetric outcomes in the compared groups. We could not find the same risk among the other immigrant women, also originating from conflict-zone countries. Several factors may influence these findings, and this study suggests that immigrant women from Somalia need more targeted care during pregnancy and childbirth.

## Background

Asian and African women have been shown to have a greater perinatal health risk than host-country women [1]. Health among refugees is mostly associated with the same factors as among the native populations, but there are some additional pre- and postmigration factors that apply to immigrants from conflict-zones, including income level in country of origin, fear of persecution, experience of torture or trauma, the asylum process, social networks, employment, and living conditions in the new country as well as acculturation and language factors [2–6].

Studies have shown an increased risk of neonatal mortality among immigrants, with refugees reported to be the most vulnerable group [7, 8]. Gagnon et al. [9, 10] found that refugees had a lower risk for emergency cesarean section (CS) relative to other immigrants in Canada; however, African women had higher CS rates, irrespective of their migration status. In Norway, Somali women in particular have been reported to have more frequent perinatal complications than ethnic Norwegians [11]. Naimy et al. [12] found that Afghan women had the highest perinatal mortality rates in Norway, with the second and third highest rates in Pakistani and Somali women, respectively. Moreover, perinatal mortality rates were lower for all immigrant groups in Norway compared with their own country of birth.

Asylum seekers and refugees have been shown to more frequently fulfill the criteria of posttraumatic stress disorder (PTSD) relative to other immigrants [3, 13]. PTSD is associated with changes in the hypothalamic-pituitary-adrenal axis [14]. Changes in cortisol levels and other stress hormones have been thought to influence pregnancy and the unborn fetus. However, conflicting results have been reported on the effect of PTSD on pregnancy outcomes such as preterm birth and low birth weight [15, 16]. Stress may also affect maternal behavior, which can ultimately lead to adverse outcomes [17].

Somalia-born women have reported experiences of widespread war-related violence, including non-partner sexual violence and intimate partner violence, before migration to Sweden [18]. The majority of immigrants with a refugee background in Norway originate from Somalia and Iraq. Afghanistan and Kosovo place in fifth and eighth place, respectively, on the list of the highest number of immigrants with a refugee background [19]. Because immigrants from conflict-zone countries most likely have experienced traumatic life events, we wanted to elucidate their obstetric outcomes relative to those of Norwegian women. Although the outcomes of Somali women are well studied in Nordic countries, women originating from Afghanistan, Iraq, and Kosovo are not as well described in the literature. In addition, the existing knowledge has not yet differentiated between levels of maternity care. The maternity ward at Baerum Hospital does not have a children’s section and is classified as a low-risk maternity ward. The women who give birth here comprise a particularly low-risk group, which includes those at more than 35 weeks of gestation and expecting a healthy baby. The aim of this study was to compare the obstetric outcomes of immigrant women originating from the conflict-zone countries of Somalia, Afghanistan, Iraq, and Kosovo with those of ethnic Norwegian women, all giving birth at the low-risk maternity ward at Baerum Hospital. We hypothesized that immigrant women from these four countries had similar risks for adverse obstetric outcomes relative to those of Norwegians because of their similar background as immigrants from conflict-zone countries.

## Methods

### Study design

This study was a population-based study with a prospective cohort design. The study period was between January 1, 2006, and December 31, 2010, and took place at the low-risk maternity ward at Baerum Hospital in Norway.

Data were extracted from information recorded during pregnancy, birth, and the early postpartum period by the Medical Birth Registry of Norway (MBRN). In addition, Statistics Norway provided information on maternal country of birth and origin and age at immigration from the Population Database and information on maternal education from the National Education Database. The MBRN identified the study participants, and a de-identified file was sent to the research team.

### Study population and setting

Baerum Hospital is located near Oslo, the capital of Norway. A pediatrician makes daily visits to the postnatal unit; otherwise, the on-call anesthetist is always available for neonatal resuscitation. The parturient women are at more than 35 weeks of gestation and expecting a healthy baby. Women with gestational diabetes and preeclampsia give birth at the hospital, but women with type 1 diabetes, preterm labor before week 35^0^, or pregnancies with more than two fetuses or fetuses with known health issues are referred to hospitals with a NICU. Sick babies are transferred to a NICU at another hospital. The postnatal unit cares for preterm newborns that have reached at least gestational week 35^0^. The maternity ward manages some high-risk deliveries but is considered a low-risk maternity ward because it has no NICU.

In Norway, all maternity services are free of charge, and each hospital has a geographical region for which it is responsible. Baerum Hospital functions as a local hospital for the suburban municipalities Asker and Baerum with a population of approximately 155,500 people. During 2009, the number of births at the maternity ward was 2583.

This study included all parturient women originating from Somalia (*n* = 278), Iraq (*n* = 166), Afghanistan (*n* = 71), Kosovo (*n* = 67), and Norway (*n* = 6826) during the study period. For women who gave birth more than once during the study period, we included only data for the first birth registered during the study period, in order to maintain independence for the included participants in the study. For twin births, the analysis included data for the first-born twins, with data for the second-born twins excluded.

### Variables

The main exposure variable was the women’s country of birth. Women classified as ethnic Norwegian were born in Norway and had two Norwegian-born parents and four Norwegian-born grandparents. The four immigrant groups were chosen because of the recent or ongoing conflicts in their home countries. The immigrant populations originating from these four countries are increasing in Norway and have not yet reached their peak [20].

### Dependent variables

The obstetric outcomes included onset of labor (categorical: spontaneous, induced, or CS), placental abruption (International Classifications of Diseases [ICD-10]: O45, dichotomous), placenta previa (ICD-10: O44, dichotomous), operative delivery (operative vaginal delivery [dichotomous]; or elective [dichotomous], or emergency CS [dichotomous]), perineal rupture (grade 3 and 4, dichotomous), episiotomy (dichotomous), postpartum bleeding >500 mL (dichotomous), epidural analgesia (dichotomous), labor dystocia (cephalopelvic disproportion, stimulated contractions by oxytocin infusion, or slow progress of labor, dichotomous), gestational age (continuous in days, gestational age was based on ultrasound findings at approximately week 18 of pregnancy or was calculated from the date of the first day of the last menstrual period), preterm birth (35^0^–36^6^ weeks, dichotomous), postterm birth (≥42^0^ weeks, dichotomous), Apgar score (<7 at 5 min, dichotomous), umbilical cord complications (dichotomous), meconium-stained liquor (dichotomous), birth weight (continuous in grams; dichotomous variables: small for gestational age [SGA]; and large for gestational age [LGA]), transfer to a NICU (dichotomous), and neonatal jaundice (dichotomous). The variables SGA and LGA are in reference to the 10^th^ and 90^th^ percentiles, respectively, for the weight-by-gestation by gender percentiles published by Skjærven et al. [21] in 2000. These curves are based on live single births in Norway with fetal age at least 20 weeks in a 12-year period from 1987–1998.

### Independent variables

Maternal background characteristics were recorded in order to examine differences in characteristics between each immigrant group relative to those of Norwegian women. In addition, many of these characteristics were used as independent variables in regression models when examining the obstetric outcomes. We recorded duration of residence in Norway before birth (continuous in years; and categorical: ≤1, 2–5, 6–10, or >10), maternal age (continuous in years), parity (categorical: 0 or ≥1), educational level (categorical: <12, or ≥12 years or undocumented), marital status (categorical: single or married/cohabiting), cigarette smoking (at the start and end of pregnancy, dichotomous), maternal health issues before pregnancy (dichotomous; if any health issues were registered, including asthma, chronic hypertension, chronic kidney disease, recurrent urinary tract infection, rheumatoid arthritis, heart condition, epilepsy, diabetes mellitus type 2, and thyroid condition), maternal health issues during pregnancy (dichotomous; if any health issues were registered, included vaginal bleeding [ICD-10: O46, also used as a dichotomous variable], hypertension, eclampsia, preeclampsia, HELLP, anemia [hemoglobin level <9 g/dL, also used as a dichotomous variable], rubella disease, venereal disease), twin birth (dichotomous).

### Statistical analysis

Categorical variables were compared using cross-tabulations with Pearson’s *χ*^2^ test or Fisher’s exact test. Some of the variables with more than two categories were tested using *χ*^2^ test for trends in order to reduce the risk of type I error occurring with multiple testing. The means of maternal age and birth weight were compared using one-way ANOVA with Bonferroni corrections. The medians of duration of residence in Norway and gestational age were compared using the Mann-Whitney *U* test. Proportions were compared between each immigrant group and the ethnic Norwegian group.

Multiple logistic regression analysis was performed to calculate odds ratios (OR) with 95 % confidence intervals (CI) and multiple linear regression analysis was performed to calculate β-coefficients for differences in birth weight in grams. The regression analyses were conducted stepwise by including more independent variables in each model. The selection of which independent variables to control for were made based on previous knowledge from an internal qualitative assurance study in addition to general obstetric knowledge [22]. Model 1 included maternal age (continuous in years) and parity (para 0 as reference). Model 2 included the variables from model 1 in addition to marital status (married/cohabiting as reference) and educational level (≥12 years as reference). Model 3 included variables from models 1 and 2 in addition to various obstetric and maternal confounders that were different for the various outcomes; twin birth (no as reference) was included in the models epidural analgesia and meconium-stained liquor; twin birth and induced labor (no as reference) were included in the model examining labor dystocia; twin birth and previous CS (no as reference) were included in the model examining emergency CS; twin birth and maternal cigarette smoking at end of pregnancy (no as reference) were included in the model examining SGA; gestational diabetes (no as reference) was included in the model examining LGA; and gestational age (continuous in days) and twin birth was included in the model examining birth weight. In the models examining the obstetric outcomes induced labor and postterm birth, model 2 was the final model. In all the models, the Norwegian women were used as a reference.

Missing values for variable educational level were coded as not documented and entered in the analyses. This variable had significantly more missing values in the immigrant groups relative to the Norwegians. There was also missing information on birth weight and gestational age in 40 participants. These participants were excluded from analyses where these values were included. The variables cigarette smoking at the start and end of pregnancy had many missing values. Proportions of smokers included only participants who were willing to provide this information to the MBRN, since women may reserve the right to not have their smoking habits on record. In the regression analysis of SGA, the variable was coded using three categories, with those with missing information coded as unknown in order to include all the participants.

The threshold for statistical significance was set at *P* < 0.01 after making Bonferroni corrections in order to reduce the risk of type I error due to multiple testing. The residuals were normally distributed in the regression model examining birth weight. Furthermore, we tested for collinearity between the exposure variable country of birth and educational level, and for interactions between the independent variables and country of origin by making interaction terms. However, no collinearity or interactions were present. The statistical analyses were conducted using IBM SPSS Statistics version 21.0 for Windows.

### Ethical consideration

Permission from the Regional Committees for Medical and Health Research Ethics, REC South East, was obtained: Ref no 2012/267.

## Results

The study included 582 immigrant women from four different conflict-zone countries and 6826 ethnic Norwegians.

### Background characteristics

Differences were found in many background characteristics between women from all four conflict-zone countries relative to those of Norwegians (Table 1). Afghan women had the shortest median length of stay (2 years, interquartile range 5) as well as the youngest mean age at 26.4 years (SD 4.9), and Norwegians had the highest mean age at 31.8 years (SD 4.5). All immigrant groups had more teenage mothers relative to the Norwegian group, with the highest proportion in the Iraqi group. Somali women were least likely to be married or cohabiting and to have a higher education. There were more multiparous women in the Somali group, four of whom (1.9 %) had experienced a previous stillbirth. No difference was found in prevalence of previous CS between immigrants and Norwegians. Somali women had fewer registered health issues before pregnancy and had the highest prevalence of anemia followed by women from Afghanistan and Iraq. Furthermore, no differences were found in the proportions of twin births.

**Table 1:** Comparison of background characteristics of immigrant women originating from four different conflict-zone countries with ethnic Norwegian women in a low-risk maternity ward in Norway (*N* = 7408)

	Norway	Somalia		Iraq		Afghanistan		Kosovo	
	*n* = 6826	*n* = 278	*P*-value*	*n* = 166	*P*-value*	*n* = 71	*P*-value*	*n* = 67	*P*-value*
Duration of residence in Norway prior birth, years, median (interquartile range)	32 (6)	6 (8)	<0.001^†^	5 (5)	<0.001^†^	2 (5)	<0.001^†^	6 (6)	<0.001^†^
≤1	–	60 (21.6)		37 (22.3)		22 (31.0)		15 (22.4)	
2–5	–	67 (24.1)		60 (36.1)		30 (42.3)		16 (23.9)	
6–10	–	87 (31.3)		47 (28.3)		16 (22.5)		23 (34.3)	
>10	6826 (100.0)	64 (23.0)		22 (13.3)		3 (4.2)		13 (19.4)	
Maternal age, years, mean (SD)	31.8 (4.5)	28.8 (6.0)	<0.001^§^	28.0 (5.7)	<0.001^§^	26.4 (4.9)	<0.001^§^	27.6 (5.9)	<0.001^§^
Teenage mothers (17–19 years)	24 (0.4)	10 (3.6)	<0.001^‡^	11 (6.6)	<0.001^‡^	3 (4.2)	0.003^‡^	3 (4.5)	0.002^‡^
Marital status			<0.001		0.003		0.408^‡^		<0.001^‡^
Married/cohabiting	6482 (95.0)	180 (64.7)		149 (89.8)		66 (93.0)		55 (82.1)	
Single	344 (5.0)	98 (35.3)		17 (10.2)		5 (7.0)		12 (17.9)	
Educational level			<0.001^a,‡^		<0.001^a,‡^		<0.001^a,‡^		<0.001^a,‡^
<12 years	1905 (27.9)	192 (69.1)		89 (53.6)		43 (60.6)		35 (52.2)	
≥12 years	4918 (72.0)	10 (3.6)		27 (16.3)		7 (9.9)		9 (13.4)	
Undocumented	3 (0.0)	76 (27.3)		50 (30.1)		21 (29.6)		23 (34.3)	
Parity			<0.001		0.030		0.972		0.017
Para 0	3543 (51.9)	72 (25.9)		72 (43.4)		37 (52.1)		25 (37.3)	
Para +1	3283 (48.1)	206 (74.1)		94 (56.6)		34 (47.9)		42 (62.7)	
Previous stillbirth^b^	12 (0.4)	4 (1.9)	0.012^‡^	0	1.000^‡^	0	1.000^‡^	0	1.000^‡^
Previous cesarean section^b^	512 (15.6)	41 (19.9)	0.101	8 (8.5)	0.061	4 (11.8)	0.540	4 (9.5)	0.280
Health issues before pregnancy^c^	1369 (20.1)	20 (7.2)	<0.001	23 (13.9)	0.048	7 (9.9)	0.032	9 (13.4)	0.177
Health issues during pregnancy^d^	999 (14.6)	46 (16.5)	0.378	21 (12.7)	0.474	6 (8.5)	0.142	3 (4.5)	0.019
Diabetes mellitus type 2 or gestational diabetes	51 (0.7)	6 (2.2)	0.023^‡^	3 (1.8)	0.136^‡^	1 (1.4)	0.417^‡^	2 (3.0)	0.094^‡^
Anemia (hemoglobin level <9 g/dL)	59 (0.9)	31 (11.2)	<0.001^‡^	7 (4.2)	0.001^‡^	4 (5.6)	0.004^‡^	0	1.000^‡^
Twin birth	59 (0.9)	2 (0.7)	1.000^‡^	3 (1.8)	0.182^‡^	0	1.000^‡^	1 (1.5)	0.455^‡^
Cigarette smoking^e^									
Start of pregnancy	692 (10.1)	7 (2.5)	<0.001	2 (1.2)	<0.001	1 (1.4)	0.015	8 (11.9)	0.627
End of pregnancy	205 (3.0)	5 (1.8)	0.245	1 (0.6)	0.097	0	0.276	6 (9.0)	0.016

Data are presented as n (%) unless indicated otherwise

*P-value is in reference to Norwegian women

^†^Mann-Whitney *U* test, ^‡^Fisher Exact test, ^§^One-Way ANOVA test, otherwise Pearson’s *χ* ^2^ test is used

^a^χ^2^ test for trends, *P*-value for all including categories

^b^Analyses include only women who were para 1 +

^c^Including any of these: Asthma, chronic hypertension, chronic kidney disease, recurrent urinary tract infection, rheumatoid arthritis, heart condition, epilepsy, diabetes mellitus, thyroid condition

^d^Registered any of these health issues during pregnancy: Bleeding, hypertension, eclampsia, preeclampsia, HELLP, anemia, rubella disease, venereal disease

^e^Those who were willing to provide information

### Obstetric outcomes

In comparison with ethnic Norwegian women, no differences were found in the proportions of placental abruption, placenta previa, perineal rupture grade 3 or 4, episiotomy, operative vaginal delivery, elective CS, postpartum bleeding >500 mL, late preterm birth (35^0^–36^6^ weeks), umbilical cord complications, transfer to a NICU, or neonatal jaundice (data not shown). Women from Kosovo did not differ from the Norwegian women in any of the obstetric outcomes tested (Table 2).

**Table 2:** Comparison of obstetric outcomes of immigrant women originating from four different conflict-zone countries compared with ethnic Norwegian women in a low-risk maternity ward in Norway (*N* = 7408)

	Norway	Somalia		Iraq		Afghanistan		Kosovo	
	*n* = 6826	*n* = 278	*P*-value*	*n* = 166	*P*-value*	*n* = 71	*P*-value*	*n* = 67	*P*-value*
Start of labor			0.034^a^		0.913^a^		0.020^a,‡^		0.138^a,‡^
Spontaneous	5596 (82.0)	216 (77.7)		137 (82.5)		67 (94.4)		53 (79.1)	
Induced labor	762 (11.2)	45 (16.2)		19 (11.4)		3 (4.2)		12 (17.9)	
Cesarean section	468 (6.9)	17 (6.1)		10 (6.0)		1 (1.4)		2 (3.0)	
Epidural analgesia	2536 (37.2)	53 (19.1)	<0.001	60 (36.1)	0.791	19 (26.8)	0.071	23 (34.3)	0.634
Labor dystocia	2918 (42.7)	95 (34.2)	0.005	74 (44.6)	0.638	31 (43.7)	0.877	29 (43.3)	0.930
Operative delivery									
Instrumental vaginal delivery	942 (13.8)	28 (10.1)	0.076	23 (13.9)	0.984	7 (9.9)	0.338	7 (10.4)	0.428
Elective cesarean section	403 (5.9)	12 (4.3)	0.269	7 (4.2)	0.361	1 (1.4)	0.128^‡^	2 (3.0)	0.436^‡^
Emergency cesarean section	583 (8.5)	38 (13.7)	0.003	16 (9.6)	0.618	4 (5.6)	0.382	5 (7.5)	0.753
Gestational age, days, median (interquartile range)^b^	282 (13)	284 (14)	0.003^†^	280 (12)	0.008^†^	277.5 (12)	0.002^†^	278 (13)	0.104^†^
Preterm (35^0^–36^6^ weeks)	174 (2.6)	7 (2.6)	1.000	5 (3.0)	0.618^‡^	0	0.265^‡^	1 (1.5)	1.000
Postterm (≥42^0^ weeks)	644 (9.5)	43 (15.9)	0.001	9 (5.4)	0.079	2 (2.8)	0.057	7 (10.4)	0.778
Meconium-stained liquor	1106 (16.2)	93 (33.5)	<0.001	34 (20.5)	0.140	13 (18.3)	0.632	10 (14.9)	0.778
Apgar score <7 at 5 min^c^	63 (0.9)	7 (2.5)	0.019^‡^	1 (0.6)	1.000	1 (1.4)	0.482^‡^	0	1.000
Birth weight, g, mean (SD)^b^	3598 (509)	3382 (550)	<0.001^§^	3397 (505)	<0.001^§^	3363 (535)	0.001^§^	3539 (541)	1.000^§^
SGA^b^	507 (7.5)	62 (23.0)	<0.001	24 (14.8)	0.001	12 (17.6)	0.003	7 (10.4)	0.345^‡^
LGA^b^	667 (9.8)	10 (3.7)	0.001	6 (3.7)	0.008	2 (2.9)	0.049	8 (11.9)	0.552

Data are presented as n (%) unless indicated otherwise

*P-value is in reference to Norwegian women

^†^Mann-Whitney *U* test, ^‡^Fisher Exact test, ^§^ One-Way ANOVA test, otherwise Pearson’s *χ* ^2^ test is used

^a^χ^2^ test for trends, *P*-value for all including categories

^b^Missing information on birthweight or gestational age on 40 participants (25 from Norway; 8 from Somalia; 4 from Iraq; and 3 from Afghanistan)

^c^Stillborn babies were excluded from analyses (*n* = 16; 12 from Norway; 1 from Somalia; 2 from Iraq; and 1 from Afghanistan)

Somali women differed from the Norwegians in proportions of several obstetric outcomes (Table 2). When adjusted for confounding factors, they were found to have reduced OR for epidural analgesia and for having an LGA infant, but increased risks for emergency CS, postterm birth, meconium-stained liquor, and having an SGA infant (Table 3). The weight difference for babies born to Somali women was −280 g (95 % CI −336 to –223). Somali women also differed in the frequency of induced labor and labor dystocia, but after adjusting for confounding variables, these associations were not statistically significant.

**Table 3:** Odds ratio of obstetric outcomes by country of origin, in reference to Norwegian women (*N* = 7408)

	n	Crude	Model 1	Model 2	Model 3
			Adjusted for maternal age and parity	Adjusted for model 1 + marital status and educational level	Adjusted for model 2 + obstetric and maternal factors
Induced labor	841 cases				
Norway	6826	Reference	Reference	Reference	
Somalia	278	1.54 (1.11–2.13)	1.92 (1.37–2.70)	1.40 (0.94–2.08)	
Iraq	166	1.03 (0.63–1.67)	1.27 (0.78–2.08)	0.97 (0.57–1.65)	
Afghanistan	71	0.35 (0.11–1.12)	0.47 (0.15–1.49)	0.36 (0.11–1.17)	
Kosovo	67	1.74 (0.93–3.26)	2.23 (1.18–4.23)	1.65 (0.84–3.24)	
Epidural analgesia	2691 cases				
Norway	6826	Reference	Reference	Reference	Reference^a^
Somalia	278	0.40 (0.29–0.54)	0.49 (0.36–0.68)	0.40 (0.28–0.56)	0.40 (0.28–0.56)
Iraq	166	0.96 (0.70–1.32)	1.01 (0.72–1.41)	0.86 (0.60–1.24)	0.85 (0.59–1.23)
Afghanistan	71	0.62 (0.36–1.05)	0.56 (0.33–0.97)	0.48 (0.27–0.84)	0.48 (0.27–0.85)
Kosovo	67	0.88 (0.53–1.47)	0.99 (0.59–1.68)	0.83 (0.48–1.44)	0.82 (0.47–1.43)
Labor dystocia	3147 cases				
Norway	6826	Reference	Reference	Reference	Reference^b^
Somalia	278	0.70 (0.54–0.89)	1.09 (0.83–1.43)	0.98 (0.72–1.33)	0.95 (0.70–1.29)
Iraq	166	1.08 (0.79–1.47)	1.36 (0.98–1.90)	1.20 (0.84–1.72)	1.20 (0.84–1.72)
Afghanistan	71	1.04 (0.65–1.66)	1.18 (0.72–1.95)	1.04 (0.62–1.75)	1.10 (0.66–1.85)
Kosovo	67	1.02 (0.63–1.66)	1.43 (0.85–2.40)	1.25 (0.73–2.14)	1.20 (0.70–2.07)
Emergency cesarean section	646 cases				
Norway	6826	Reference	Reference	Reference	Reference^c^
Somalia	278	1.70 (1.19–2.41)	2.63 (1.82–3.80)	1.90 (1.24–2.89)	1.81 (1.17–2.80)
Iraq	166	1.14 (0.68–1.93)	1.53 (0.90–2.61)	1.26 (0.71–2.22)	1.34 (0.75–2.38)
Afghanistan	71	0.64 (0.23–1.76)	0.88 (0.32–2.45)	0.71 (0.25–2.01)	0.74 (0.26–2.11)
Kosovo	67	0.86 (0.35–2.16)	1.22 (0.48–3.09)	0.98 (0.38–2.53)	0.98 (0.37–2.62)
Postterm birth^g^	703 cases				
Norway	6801	Reference	Reference	Reference	
Somalia	270	1.81 (1.30–2.54)	2.29 (1.62–3.24)	1.93 (1.29–2.90)	
Iraq	162	0.50 (0.24–1.02)	0.59 (0.29–1.22)	0.55 (0.26–1.16)	
Afghanistan	68	0.29 (0.07–1.19)	0.35 (0.09–1.44)	0.33 (0.08–1.37)	
Kosovo	67	1.12 (0.51–2.45)	1.38 (0.62–3.05)	1.24 (0.54–2.82)	
Meconium-stained liquor	1256 cases				
Norway	6826	Reference	Reference	Reference	Reference^a^
Somalia	278	2.60 (2.01–3.36)	2.93 (2.25–3.83)	2.40 (1.76–3.26)	2.39 (1.76–3.25)
Iraq	166	1.33 (0.91–1.95)	1.44 (0.98–2.13)	1.22 (0.81–1.85)	1.23 (0.81–1.86)
Afghanistan	71	1.16 (0.63–2.12)	1.26 (0.68–2.32)	1.06 (0.56–1.98)	1.05 (0.56–1.96)
Kosovo	67	0.91 (0.46–1.78)	1.00 (0.51–1.98)	0.84 (0.42–1.69)	0.84 (0.42–1.70)
SGA^g^	612 cases				
Norway	6801	Reference	Reference	Reference	Reference^d^
Somalia	270	3.70 (2.75–4.98)	4.57 (3.33–6.26)	3.50 (2.43–5.04)	3.97 (2.73–5.77)
Iraq	162	2.16 (1.39–3.36)	2.37 (1.51–3.74)	2.05 (1.27–3.32)	2.21 (1.36–3.60)
Afghanistan	68	2.66 (1.42–4.99)	2.78 (1.46–5.29)	2.41 (1.24–4.68)	2.77 (1.42–5.39)
Kosovo	67	1.45 (0.66–3.18)	1.64 (0.74–3.64)	1.37 (0.60–3.10)	1.32 (0.58–3.03)
LGA^g^	693 cases				
Norway	6801	Reference	Reference	Reference	Reference^e^
Somalia	270	0.35 (0.19–0.67)	0.31 (0.17–0.60)	0.33 (0.16–0.66)	0.32 (0.16–0.64)
Iraq	162	0.35 (0.16–0.80)	0.36 (0.16–0.81)	0.35 (0.15–0.84)	0.35 (0.15–0.83)
Afghanistan	68	0.28 (0.07–1.14)	0.31 (0.08–1.27)	0.30 (0.07–1.27)	0.30 (0.07–1.25)
Kosovo	67	1.25 (0.59–2.62)	1.25 (0.59–2.66)	1.27 (0.56–2.89)	1.23 (0.54–2.80)
Birth weight^g,h^					
Norway	6801	Reference	Reference	Reference	Reference^f^
Somalia	270	−216 (−278 to –154)	−254 (−317 to –192)	−218 (−285 to –150)	−280 (−336 to –223)
Iraq	162	−201 (−280 to –121)	−213 (−292 to –133)	−197 (−279 to –116)	−170 (−238 to –102)
Afghanistan	68	−235 (−357 to –113)	−231 (−352 to –109)	−220 (−343 to –97)	−150 (−252 to –47)
Kosovo	67	−59 (−182 to 64)	−78 (−200 to 44)	−55 (−179 to 69)	−45 (−148 to 58)

Data is presented as odds ratio and 95 % confidence interval (CI) unless indicated otherwise

Obstetric or maternal factors

^a^twin birth

^b^twin birth and induced labor

^c^twin birth and previous CS

^d^twin birth and cigarette smoking at end of pregnancy

^e^gestational diabetes

^f^gestational age and twin birth

^g^Missing information on birth weight or gestational age reduces the n by 40 in this model (25 from Norway; 8 from Somalia; 4 from Iraq; and 3 from Afghanistan), *N* = 7368

^h^Linear regression showing β (95 % CI)

Women from Iraq and Afghanistan differed in median gestational age (a few days shorter relative to Norwegians), mean birth weight, and in proportion of SGA infants. When adjusted for maternal age, parity, marital status, educational level, twin birth, and gestational age, the weight difference was −170 g (95 % CI −238 to –102) and −150 g (95 % CI −252 to –47) for babies born to women from Iraq and Afghanistan, respectively (Table 3). Their OR of having an SGA infant was 2.21 (95 % CI 1.36–3.60) and 2.77 (95 % CI 1.42–5.39), respectively, after adjusting for confounding factors. In addition, women from Iraq had a reduced OR of having an LGA infant with an adjusted OR of 0.35 (95 % CI 0.15–0.83) (Table 3).

Socioeconomic status, measured by educational level and marital status, affected the estimates greatly when assessing associations stepwise as shown in Table 3. Estimates were weakened the most when adding these variables into the regression models.

## Discussion

This is the first study examining immigrants from countries considered conflict-zones in a low-risk maternity ward. We observed some differences in obstetric outcomes between immigrant women from conflict-zone countries and ethnic Norwegians. Somali women were most at risk for adverse obstetric outcomes, while women from Iraq and Afghanistan differed from the Norwegians in gestational age, having an SGA infant, and mean birth weight. Women from Kosovo did not differ from the Norwegians in any of the obstetric outcomes that were tested, which is consistent with previous findings from a case-control study from London [23]. Hence, our hypothesis, that immigrant women from these four countries had similar risks for adverse obstetric outcomes relative to those of Norwegians because of their similar background as immigrants from conflict-zone countries, was not confirmed. However, the group of women from Somalia was larger than the other three immigrant groups; hence, the possibility of more accurately estimating the associations is more likely for this group. Furthermore, these women could have different experiences of war and conflict, and the duration of conflict may vary between these four countries. We made additional analyses of the association between duration of residence in Norway and risks of obstetric outcomes, but no such associations were found. Our observations of Somali women’s increased risk of adverse obstetric outcomes are consistent with previous studies of the total Norwegian birth cohort, and in other western countries [1, 11, 24–26].

Being an immigrant from a conflict-zone country does not necessarily mean that you have been exposed to trauma or have the same experiences that could influence obstetric outcomes. However, United States research has documented a high level of torture experience (47 %) among Somali immigrant women [27]. Byrskog and collaborators [18] explored immigrant Somali women’s experiences of war and violence before migration to Sweden and discovered that the actions of war had created fear, experiences of loss, and separation from family. Additional research in Sweden has revealed that foreign-born women from middle/low-income countries reported a greater exposure to perceived threats of violence and physical violence relative to Swedish-born women [28] and this was largely explained by background factors such as household income. Unfortunately, we have not found other studies reporting the prevalence of trauma and torture among immigrant women from Iraq, Afghanistan, and Kosovo.

A recent Swedish cohort study investigated preterm birth in war refugees and found an association between a short duration of stay in Sweden and preterm birth [29]. In our study, in the low-risk maternity ward, examining preterm birth was limited to those giving birth between 35^0^ and 36^6^ weeks of gestation. None of the immigrant groups showed an increased risk of preterm birth, on the contrast, Somali women displayed an increased risk of postterm birth, which is consistent with reports from Australia [30] and the United States [25]. These findings are, however, contradictory to what we would expect since these women originate from a country with long-lasting war and conflict. An answer to this puzzle could be that there is no correlation between Somali women’s experiences of war and our expectations of their higher risk of developing mental health problems because of these experiences. Råssjö and colleagues [31] reported almost none mental health problems in Somali women in Sweden. This may be due Somali women’s way of handling challenges described by Byrskog et al. [18] were these expressed that they had to accept the situation, look forward, and not dwell on what cannot be changed. Because of their difficulties, the Somali women had learned to be strong.

Another aspect in discussing Somali women’s increased risk of postterm birth is their attendance to antenatal care. In Sweden, Somali women attend antenatal care later in pregnancy and have fewer antenatal care visits compared with Swedish controls [31]. Furthermore, many foreign-born women in Sweden make unplanned visits to delivery wards, and as a consequence, miss out on the full benefits of planned routine antenatal care [32]. We can speculate that perhaps the timing of ultrasound screening is inadequate and that the estimation of gestational age is uncertain for these women. Early ultrasound estimation is a better estimate than menstrual cycles [33]. Women are routinely invited for ultrasound screening at approximately week 18 of pregnancy at our hospital, assuming they have visited the doctor or midwife who has sent a referral to the hospital.

A study performed in Basrah governorate, Iraq, in 1983, reported a mean birth weight of 3.32 ± 0.41 kg and a 3.6 % prevalence of low birth weight [34]. This is in line with the findings in our study. The mean birth weight of Afghan children in Afghanistan has been reported as 3174.7 g (SD 455.8) [35], which is lower than the findings in our low-risk maternity unit, possibly because our study participants were more than 35^0^ weeks gestation. Malin and Gissler [26] reported that 4.7 % of women of Somali origin and 3.5 % of women originating from Iran, Iraq, and Afghanistan experienced giving birth to an SGA infant in Finland. Råssjö et al. [31] reported that 8.1 % of Somali women in their study in Sweden experienced the same. These proportions are much lower than the ones in our study. However, different weight-by-gestation curves are used in calculating percentiles in the various countries depending on their own birth population. In addition, different definitions are used in classifying SGA infants. The validity of the curve used in our study can also be discussed as Boshari et al. [36] discovered that birth weight percentiles among term infants born to immigrants in Canada were higher relative to those infants born to mothers in their respective native countries. Moreover, Urquia et al. [37] assessed the classification of infants who are considered SGA and LGA using a standard Canadian birth weight curve, in addition to a curve tailored to maternal global region of origin. They discovered that the latter curve appeared more appropriate for assessing risk of adverse outcomes in infants classified as SGA and LGA born to immigrant mothers, particularly those who originated from East and South Asia. It is therefore time to develop a more customized birth weight curve in Norway, which takes maternal height and country of birth into consideration. This could reduce the number of infants considered SGA; moreover, it could be used to identify more infants who are considered LGA and in need of special attention. However, the proportions of SGA infants were much higher for the immigrants from Somalia, Iraq, and Afghanistan relative to the Norwegians. Women from Somalia had the highest proportion and nearly four times increased OR relative to the Norwegians. Qualitative studies in Scandinavia have reported that many Somali women limit their food intake during pregnancy from fear of delivering a large baby [38, 39]. A higher proportion of Somali women also had anemia in our study. Perhaps some of them restricted their diet and became anemic as a consequence, which might contribute to some of the increased risk of having an SGA infant in our study. Proper antenatal care including good communication can enhance women’s knowledge and more likely create a trusting relationship [40]. Broken trust in maternal care providers can result in late entry into antenatal care, low adherence to recommendations, and inappropriate decision-making [41]. Enhancing Somali women’s knowledge could possibly help reduce the practice of restricting their diet and ultimately reduce the risk of having SGA infants.

Somali women’s high proportion of anemia might explain some of their increased risk for adverse obstetric outcomes in our study. However, we cannot exclude the possibility of poor healthcare, since suboptimal perinatal care was significantly higher among African immigrants in Sweden who experienced perinatal deaths [42]. Suboptimal antenatal and obstetric care among non-Western women who had increased risk of stillbirth was also found in Norwegians [43]. Non-Western women were less likely to attend the antenatal program and to follow recommendations. In addition, inadequate communication was found in almost half of the cases. Furthermore, suboptimal care factors have been reported to be present in maternal deaths in Sweden, with those related to immigrant women was poor communication, lack of professional interpreters, and noncompliance with healthcare workers advice [44]. Language and cultural barriers may create misunderstandings that can negatively affect treatments and reduce access to proper healthcare services when patients cannot express their needs [45, 46]. Using an interpreter may reduce the likelihood of an adverse pregnancy outcome [47]. National and international standards state that interpreting is the healthcare worker’s communicative responsibility [45]. Interpreters, however, are not used during labor in our maternity ward. The low proportion of women from Somalia having epidural analgesia can also be related to poor care or poor communication. However, this observation is also consistent with previous findings [24]. Interview studies with women originating from Somalia have revealed that they fear interventions during labor, and perhaps that is the reason for their low use of this analgesia [38, 39]. Nonetheless, proper information during pregnancy and good communication throughout pregnancy and labor could possibly change their opinion of this intervention. Unfortunately, information on the immigrants’ participation in the antenatal program or on women’s linguistic challenges could not be obtained in our study.

Socioeconomic factors can also influence obstetric outcomes [48]. If we combine the results of the two variables educational level and marital status in our study, Somali women had the lowest socioeconomic status with the highest proportions of single and poorly educated women in our study relative to Norwegians. These were also the variables that affected the estimates the most in the regression models. Dejin-Karlsson et al. [49, 50] in Sweden reported on a stress hypothesis, which implied that psychosocial factors influenced intrauterine growth. They reported an increased risk of SGA infants for women with low social stability, social participation, emotional support, and instrumental support (access to advice and information). Psychosocial factors were reported to be more important risk factors for having SGA infants among immigrant mothers relative to mothers of Swedish origin [50]. An increased duration of residence in the new country has been shown to increase the immigrants’ experience of having social support during pregnancy and to not increase the risk of having an SGA infant in Canada [51, 52]. We examined the association between length of residence in Norway and adverse obstetric outcomes in our study. However, no associations were found. The relatively small sample size in our study could possibly explain this, and further research is needed on larger samples.

This study has methodological limitations that must be addressed. Country of birth was used as refugee background. However, immigrants originating from the present four countries are all on the list of highest number of immigrants with refugee background in Norway [19]. Information on women’s mental health issues, female genital mutilation, antenatal care attendance, and need for an interpreter was not available for this study. This information could be useful in distinguishing effects. Furthermore, the MBRN does not have information on maternal weight and height for the study population. This information could modify the results somewhat as we know from earlier work with an internal quality assurance study [22],

Most of the outcomes tested were binary variables, and larger samples are needed to detect a difference relative to continuous variables. That is perhaps why we detected differences in gestational age and birth weight, but not in other outcomes we tested for women originating from Iraq and Afghanistan. Our findings should therefore be interpreted with caution and cannot be generalized to a Norwegian population.

When examining obstetric outcomes of refugees, the most common outcome has been preterm birth. The population for this study was limited to those giving birth past 35 weeks of gestation, and expecting a healthy baby. However, other outcomes are also of interest, particular at this low-risk maternity ward. This population can be well suited to examine differences between immigrants and host-country populations, because many confounding factors (e.g., severe maternal pre-pregnancy morbidity and fetal complications) are already eliminated. The differences observed can therefore possibly be more related to maternity care received and maternal health behavior, rather than pre-pregnancy conditions. However, there can also be differences in other outcomes (e.g., preterm birth and congenital malformations) that cannot be studied at this hospital.

Furthermore, this study has an accurate registration of country of birth and origin. Another strength may be that the study was conducted at a single hospital, where women are most likely to have received the same treatment, guided by the same policies and procedures at the hospital.

Cultural and social aspects may also contribute to explaining the elevated risks for adverse obstetric outcomes among Somali women in our study, and unfortunately, our study has not been able to explore them all. Future studies should include language competency to explore the effect of communication barriers on obstetric outcomes. Research suggests that the maternity care provided for refugees needs to be adjusted to comprise continuity, quality interpreters, educational strategies, and psychosocial support [53]. Since there is now compelling evidence of Somali women’s consistent higher risk of adverse obstetric outcomes future studies should also focus on interventions for Somali women aiming at improving antenatal care attendance and enhancing their knowledge of health behavior.

## Conclusion

We found that even in our low-risk maternity ward, Somali women were most at risk for adverse obstetric outcomes. We could not find the same risk among other immigrant women, also originating from conflict-zone countries. Several factors may influence these findings, such as their antenatal care, nutrition, cultural preferences, language skills, and socioeconomic status. This study suggests that immigrant women from Somalia need more targeted care during pregnancy and childbirth, even in a low risk setting. More use of interpreters and culturally sensitive models of care should be introduced in primary care and in hospitals.

## Abbreviations

BMI: Body mass index
CI: Confidence interval
CS: Cesarean section
ICD-10: International Classifications of Diseases
LGA: Large for gestational age
MBRN: Medical Birth Registry of Norway
NICU: Neonatal intensive care unit
OR: Odds ratio
PTSD: Posttraumatic stress disorder
SGA: Small for gestational age
